# Foliar spraying of *Bradyrhizobium* and *Anabaena* improve stress tolerance under deficit irrigation of Cowpea

**DOI:** 10.1038/s41598-026-54968-1

**Published:** 2026-06-05

**Authors:** Abdelgawad Y. Elsadany, Sahar El-Nahrawy

**Affiliations:** 1 Cyanobacteria Lab., Microbiology Dept., Agric. Res. Center, Sakha Agricultural Research Station, Soils, Water and Environment Research Institute, Giza, Egypt; 2 Agricultural Research Center, Soil microbiology Research Department, Soils, Water and Environment Research Institute, Giza, 12112 Egypt

**Keywords:** *Bradyrhizobium*, *Anabaena cylindrica*, Bioactive compounds, Foliar application, Cowpea productivity, Biotechnology, Physiology, Plant sciences

## Abstract

Water deficit is considered one of the most significant factors limiting cowpea productivity, necessitating sustainable strategies to improve crop tolerance to drought stress. This study investigated the effects of foliar application of *Bradyrhizobium* and *Anabaena* on the growth, physiological performance, and productivity of cowpea under different irrigation intervals. Under the 15-day irrigation interval (water-stressed condition), the combined *Bradyrhizobium* and *Anabaena* treatment increased total seed yield by more than 30% compared with the stressed untreated control. This confirms the strong role of the consortium in improving drought tolerance and productivity under limited irrigation. *Anabaena* was identified as *Anabaena cylindrica* using *rpoC1* gene sequence analysis. Phytohormone content of *A*. *cylindrica* was quantified by HPLC and bioactive compounds from *Bradyrhizobium* sp. and *A*. *cylindrica* were detected by GC- MS. The study evaluated nodule number, leaf N, K, Na contents, K/Na ratio, chlorophyll, carotenoids, total soluble sugars (TSS), relative water content (RWC), proline concentration, antioxidant enzymes (APX and CAT), and soil enzyme activities (dehydrogenase and urease) along with yield components and total seed yield. Results showed that the consortium treatment significantly improved chlorophyll, carotenoids, TSS, and RWC, while reducing proline accumulation compared with stressed control. It also increased root nodulation, enhanced N and K uptake, reduced Na accumulation, and improved K/Na ratio. It stimulated CAT and APX activities in leaves and enhanced soil enzyme activities, leading to improved yield components and overall productivity under drought stress. These findings highlight the potential of combining *Bradyrhizobium* and *Anabaena cylindrica* as an eco-friendly and sustainable biostimulant strategy to enhance cowpea productivity and drought resilience under water-limited conditions.

## Introduction

Cowpea is a nutritionally and agronomically important legume owing to its high protein content and substantial economic value^1^. Nevertheless, cowpea productivity is considered highly vulnerable in the face of water scarcity, especially under deficit irrigation conditions, where prolonged irrigation intervals adversely affect plant growth, physiological development, and yield. Water shortage during irrigation remains one of the most significant constraints affecting agricultural production sustainability globally. Climate change, increasing competition for water resources, and rising evapotranspiration rates have exacerbated drought stress^2^. Water deficit stress negatively affects plant water relations, photosynthesis, nutrient uptake, and increases the level of reactive oxygen species (ROS), resulting in reduced plant growth and yield loss^3^. Consequently, the search for reliable and environmentally friendly strategies to improve crop tolerance under limited water availability has become a major priority in sustainable agriculture.

Microbial biostimulants have emerged as promising tools for alleviating the adverse effects of water-deficit stress^4^. Notably, plant-growth promoting rhizobacteria (PGPR) and cyanobacteria can enhance nutrient acquisition, stimulate phytohormone production, and activate antioxidant defense systems, thereby improving plant tolerance to drought stress. *Bradyrhizobium* sp. is well known for forming symbiotic associations with leguminous plants, thereby contributing to biological nitrogen fixation. Also, this bacterium can produce phytohormones and organic acids involved in regulating root morphogenesis and enhancing plant stress tolerance^5,6^. *Anabaena* sp. is another important microorganism with a wide range of potential applications due to the multifunctional role in agricultural ecosystems. It is capable of biological nitrogen fixation, nutrient solubilization, and production of several bioactive compounds, including phytohormones, antioxidants, amino acids, and phenolic compounds^7^. Biomass from *Anabaena cylindrica* has also shown effective improvement for plant growth and nutrient contents, as well as tolerance to abiotic stress conditions like drought and salinity, as a result of improvements in water relations, photosynthesis rates, as well as antioxidant enzyme activities^8^.

Foliar application of microbial extracts is one of the most practical and efficient methods for delivering bioactive compounds directly to plant tissues, overcoming soil-related limitations during water-deficit conditions. Although *Bradyrhizobium* is associated with the root zone, foliar spraying of its extract enables microbial-derived bioactive compounds to reach leaf tissues directly, bypassing the drought-induced limitations in root activity and nutrient uptake, while enhancing stress tolerance. Several reports have showed improvements in relative water content, chlorophyll content and stability, osmotic adjustment, and antioxidant capacity of various crops under drought stress upon their foliar spraying with extracts of microalgae^9,10^. The combined use of rhizobia and cyanobacteria may exert synergistic effects through integrating biological nitrogen fixation with enhanced physiological and biochemical stress resilience.

Although the large number of published studies on microbial biostimulants, information on the combined foliar application of *Bradyrhizobium* and *Anabaena* under deficit irrigation conditions in cowpea remains limited. In particular, the underlying physiological, biochemical, and soil enzymatic mechanisms for their possible synergistic effects under an extended irrigation interval are poorly understood. The present study was thus performed to investigate individual and combined applications of *Bradyrhizobium* sp. and *Anabaena cylindrica* through foliar application on growth, physiological responses, antioxidant defense system, soil enzymatic activities, mineral nutrient status, and productivity of cowpea under varying irrigation intervals. We tested the hypothesis that the combined application of *Bradyrhizobium* and *Anabaena* would enhance cowpea tolerance to water deficit by improving its water relations, photosynthetic efficiency, nutrient balance, and antioxidant capacity, thus leading to improved productivity under reduced irrigation conditions.

## Materials and methods

### Microorganisms and culture conditions

*Bradyrhizobium* sp*.* TAL – 169 and *Anabaena* sp. were obtained from Bacteriology Lab. and Algae Lab., respectively, at Sakha Agricultural Research Station, Kafr El-Sheikh, Egypt. *Bradyrhizobium* sp. was cultured on yeast extract mannitol agar (YEMA) medium until it reached the late exponential phase (≈10⁸–10⁹ CFU mL^−1^). To preserve both bacterial cells and extracellular metabolites, the culture was used as a crude microbial suspension without centrifugation^11^. *Anabaena* sp. was grown on modified Watanabe medium as described by El-Nawawy et al.^12^ One kilogram fresh biomass of *Anabaena* was collected and washed with distilled water. The biomass was homogenized using a blender for 5 minutes, and followed by further homogenization in a mortar to ensure complete cell disruption. The resulting liquid was filtered through a cotton cloth, and the filtrate was diluted with distilled water to a final volume of 1 L to obtain the stock extract. The extract was stored at 4°C and used within 24 hours^13^. The two cultures were mixed at a 1:1 (v/v) ratio immediately prior to application to ensure equal contribution of bacterial and cyanobacterial components. For foliar application, the combined microbial suspension was diluted with tap water at a ratio of 1:100 (v/v) (equivalent to 2.38 L of mixed culture per 238 L water ha^−1^). Foliar spraying was performed using a hand-held sprayer in the early morning to minimize UV exposure, evaporation, and potential wash-off losses, thereby improving microbial persistence on leaf surfaces. Applications were performed at 15, 30, and 45 days after sowing.

### GC-MS and total hormones determination

#### GC-MS determination

The collected *Bradyrhizobium* and *A. cylindrica* cells were centrifuged at g ×11.180 for 3 minutes. The pellets were collected and air-dried after filtration through Whatman No. 4 filter paper. One gram of dried biomass was extracted with 10 mL methanol, which was then centrifuged at g ×11.180 for 10 minutes. A 0.5 μm membrane filter was used to filter the supernatant to remove residual debris. After methanol evaporation, the residue was re-dissolved in dimethyl sulfoxide (DMSO). Before analysis, the extracts were kept in sterile glass vials at 4 °C in the dark^14^. A Trace GC 1310-ISQ mass spectrometer (Thermo Scientific, Austin, TX, USA) with a TG-5MS direct capillary column (30 m × 0.25 mm i.d., 0.25 μm film thickness) was used for the phytochemical analysis. The temperature schedule for the oven was programmed to begin at 40 °C, ramp up to 120 °C at a rate of 3 °C per minute, and then climb by 5 °C per minute to 280 °C, which was held for one minute. The injector and MS transfer line were maintained at 250 °C and 260 °C, respectively. Helium was used as the carrier gas at a constant flow rate of 1 mL/min. An AS1300 autosampler operating in split mode was used to automatically inject a 1 μL aliquot of the diluted extract. The solvent delay time was set to 3 min. In full scan mode, electron ionization (EI) mass spectra were captured at 70 eV over a mass range of m/z 40–1000. The temperature of the ion source was kept constant at 200 °C. By comparing retention times and mass spectra with those found in the WILEY 09 and NIST 11 mass spectral databases, compounds were tentatively identified. GC-MS analysis was conducted only for the identification of compounds in the methanolic extract and does not necessarily represent the composition of the aqueous extract used for foliar application under field conditions Huwaimel et al^15^

##### Total hormones determination

HPLC was used to identify the plant hormones (cytokinins, gibberellic acid, and indole-3-acetic acid) in the extract of *A. cylindrica*^16^. Samples were collected from 15- day- old cultures. Three biological replicates were analyzed, with each sample measured in triplicate. HPLC analysis was carried out under isocratic conditions using a C18 reversed-phase column (150 × 4.6 mm i.d.,5 µm) with a mobile phase composed of acetonitrile and acidic water (0.01% H₃PO₄) in a 60:40 (v/v) ratio. The Chromatographic separation was performed at room temperature at a flow rate of 1 mL min^−1^ using a 20 µL injection volume. Quantitative analysis was performed at 206 nm.

### *rpoC1* analysis

#### PCR reactions

The plastidial rpoC1 region was used for DNA barcoding analysis of *Anabaena* sp. PCR amplification was performed in a total reaction volume of 50 μL containing 25 μL of 2× Master Mix (OnePCR™, GeneDireX, Inc., Taipei, Taiwan), 3 μL of DNA template (10 ng μL^−1^), 2 μL of each primer (10 pmol μL^−1^), and 18 μL of nuclease-free water.. Primers code and sequence used for *rpoC1* amplification are presented in Table 1. Molecular identification of *Anabaena* sp. was performed using *rpoC1* gene barcoding analysis according to the protocol of Gugger et al. (2002)^17^.

**Table 1:** The primers name and sequence of rpoC1.

Primer code	Sequence (5’–3’)	Product size
*rpoC1-F*	GGCAAAGAGGGAAGATTTCG	600bp
*rpoC1-R*	CCATAAGCATATCTTGAGTTGG	

#### Thermo-cycling PCR program

PCR amplification was performed using a Perkin-Elmer/GeneAmp® PCR System 9700 (PE Applied Biosystems). The thermal cycling conditions consisted of an initial denaturation step at 94 °C for 5 min, followed by 40 cycles of denaturation at 94 °C for 30 s, annealing at 48 °C for 30 s, and extension at 72 °C for 1 min. A final extension step was carried out at 72 °C for 7 min.

#### Finding the PCR Products

The amplified PCR products were separated by electrophoresis on a 1.5% agarose gel containing ethidium bromide (0.5 μg mL^−1^) in 1× TBE buffer at 95 V. A 100 bp DNA ladder was used as the molecular size marker. The PCR products were visualized under UV light and photographed using a Gel Documentation System (BIO-RAD 2000).

#### PCR product purification

All PCR-amplified products were purified using an EZ-10 spin column purification kit. The PCR reaction mixture was transferred into a 1.5 mL microcentrifuge tube, and three volumes of Binding Buffer 1 were added. The mixture was then transferred to the EZ-10 column, incubated at room temperature for 2 min, and centrifuged according to the manufacturer’s instructions. Subsequently, 750 μL of wash solution was added to the column, followed by centrifugation at 10,000 rpm for 2 min to remove residual contaminants. The column was then transferred to a clean 1.5 mL microcentrifuge tube, and 50 μL of elution buffer was added. After incubation at room temperature for 2 min, the purified DNA was collected by centrifugation and stored at −20 °C until further analysis.

#### Analysis of matk and rpoC1 sequencing

The PCR products were sequenced using BigDye™ Terminator Cycle Sequencing Kits and an ABI PRISM 3730XL automatic sequencer according to the manufacturer’s instructions. The forward primer was used for single-pass sequencing of each template. Fluorescently labeled fragments were purified from unincorporated terminators using the ethanol precipitation method. After re-suspension in distilled water, the samples were electrophoresed using an ABI 3730XL sequencer (Microgen Company). The obtained sequences were analyzed using the BLAST tool available at the National Center for Biotechnology Information (NCBI). Sequence alignment was performed using Nucleotide BLAST.

### Experimental setup and treatments

During two consecutive summer growing seasons (2022 and 2023), a field experiment was conducted at Sakha Agricultural Research Station, Kafr El-Sheikh Governorate, Egypt, to evaluate the effects of foliar application of *Bradyrhizobium* sp. and *Anabaena* sp., applied individually or in combination, under different irrigation intervals on vegetative growth, physiological traits, soil biological activity, antioxidant enzyme activity, mineral nutrient content, and yield of cowpea (*Vigna unguiculata* L., cv. Kafr El-Sheikh 1). The experiment was arranged in a split-plot design with three replicates. Irrigation intervals constituted the main plots, with three irrigation regimes applied at 10, 15, and 18- day intervals from sowing. Foliar spray treatments were assigned to the sub-plots, each measuring 42 m²(10 rows, 7 mlong, with 0.70 mrow spacing). The foliar treatments included: (i) water spray (control), (ii) *Bradyrhizobium* sp. extract (1:100, v/v), (iii) *Anabaena cylindrica* extract (1:100, v/v), and (iv) a combined treatment of *Bradyrhizobium* sp. + *A. cylindrica*. Experimental treatments and their corresponding codes found in Table 2. The physical and chemical properties of the experimental soil, including, cations, anions, and available macronutrients are presented in Table 3 and 4. Nitrogen fertilization was applied at a rate of 1000 gha^−1^ using Okaden, a commercial biofertilizer formulated as a peat-based inoculum with a sterilized carrier. The inoculum was thoroughly mixed with cowpea seeds prior to sowing. Phosphorus fertilizer was applied at 480 kgha^−1^ as calcium superphosphate (15.5% P₂O₅), while potassium fertilizer was supplied at 120 kgha^−1^ as potassium sulfate (48% K₂O).Cowpea seeds were obtained from the Horticultural Research Institute, Sakha, Kafr El-Sheikh, Egypt. Seeds were sown at a rate of 75 kgha^−1^ on April 7 and April 5 during the 2022 and 2023 seasons, respectively. Seedlings were thinned to two plants per hill before the first irrigation. Physiological and biochemical measurements were conducted at the pod filling stage.

**Table 2:** Experimental treatments and corresponding irrigation intervals.

Code	Treatment	Irrigation
T_1_	Control (water spray)	Every 10 days
T_2_	Control (water spray)	Every 15 days
T_3_	Control (water spray)	Every 18 days
T_4_	*Bradyrhizobium* sp.	Every 10 days
T_5_	*Bradyrhizobium* sp*.*	Every 15 days
T_6_	*Bradyrhizobium* sp*.*	Every 18 days
T_7_	*Anabaena cylindrica*	Every 10 days
T_8_	*Anabaena cylindrica*	Every 15 days
T_9_	*Anabaena cylindrica*	Every 18 days
T_10_	Consortium (*Bradyrhizobium* sp*. + Anabaena cylindrica*)	Every 10 days
T_11_	Consortium (*Bradyrhizobium* sp*. + Anabaena cylindrica*)	Every 15 days
T_12_	Consortium (*Bradyrhizobium* sp*. + Anabaena cylindrica*)	Every 18 days

**Table 3:** Soil chemical and physical properties during the seasons 2022 and 2023.

Season	Soil chemical properties			
	pH (1:2.5)	EC (dS m^−1^)	OM (%)	ESP (%)
2022	8.12 ± 0.01	7.23 ± 0.03	1.46 ± 0.02	20.27 ± 0.11
2023	8.08 ± 0.02	7.21 ± 0.02	1.55 ± 0.03	20. 12 ± 0.22
	Soil physical properties			
	Sand	Silt	Clay	Texture
2022	27.22 ± 1.16	23.01 ± 1.13	49.77 ± 2.05	Clayey
2023	27.76 ± 1.58	23.69 ± 1.12	48.55 ± 2.01	Clayey

Values are presented as mean ± standard deviation. EC = electrical conductivity measured in the soil paste extract; OM = organic matter; ESP = exchangeable sodium percentage.

**Table 4:** Cations, anions, macronutrients of soil during the seasons 2022 and 2023.

Season	Cations (meq L^−1^)			
	Ca^+2^	Mg^+2^	Na^+^	K^+^
2022	7.22 ± 0.46	5.17 ± 1.22	22.02 ± 2.19	0.39 ± 0.06
2023	8.01 ± 0.31	5.18 ± 1.38	24.17 ± 2.09	0.35 ± 0.08
	Anions (meq L^−1^)			
	CO_3_^−2^	HCO_3_^−^	Cl^−^	SO_4_^−2^
2022	-	3.01 ± 0.55	19.00 ± 1.29	12.80 ± 2.11
2023	-	3.88 ± 0.37	21.03 ± 1.86	12.38 ± 2.02
	Available macronutrients (mg kg^−1^)			
	N	P	K	
2022	8.31 ± 0.81	7.02 ± 1.22	302 ± 17.03	
2023	9.59 ± 0.25	7.36 ±1.42	14.01	

Values are presented as mean ± standard deviation

#### Number and dry weight of nodules

At 50 days after sowing, the number of nodules per cowpea plant was recorded, and nodule dry weight (g plant^−1^) was determined using an electronic balance. Nodules were carefully detached from the roots, washed with distilled water to remove adhering soil particles. The nodules were then oven-dried at 70 °C to a constant weight and weighed using an electronic balance.

#### Photosynthetic pigments

Total chlorophyll content (mg g^−1^ FW) and carotenoids (µg g^−1^ FW) were determined according to the method described by Lichtenthaler (1987)^18^.

#### Total soluble sugars (TSS)

Total soluble sugars (TSS, µg g^−1^ FW) were determined according to the method described by Hendrix (1993)^19^.

#### Relative leaf water content (RLWC)

Relative leaf water content (RLWC) was determined according to the method described by Barrs and Weatherley (1962)^20^ and expressed as a percentage (%). RLWC was calculated using the following equation:

$$\mathbf{R}\mathbf{L}\mathbf{W}\mathbf{C}\left(\mathbf{\%}\right)=\frac{{\boldsymbol{F}}{\boldsymbol{r}}{\boldsymbol{e}}{\boldsymbol{s}}{\boldsymbol{h}}{\boldsymbol{w}}{\boldsymbol{e}}{\boldsymbol{i}}{\boldsymbol{g}}{\boldsymbol{h}}{\boldsymbol{t}}-{\boldsymbol{D}}{\boldsymbol{r}}{\boldsymbol{y}}{\boldsymbol{w}}{\boldsymbol{e}}{\boldsymbol{i}}{\boldsymbol{g}}{\boldsymbol{h}}{\boldsymbol{t}}}{{\boldsymbol{T}}{\boldsymbol{u}}{\boldsymbol{r}}{\boldsymbol{g}}{\boldsymbol{i}}{\boldsymbol{d}}{\boldsymbol{w}}{\boldsymbol{e}}{\boldsymbol{i}}{\boldsymbol{g}}{\boldsymbol{h}}{\boldsymbol{t}}-{\boldsymbol{D}}{\boldsymbol{r}}{\boldsymbol{y}}{\boldsymbol{w}}{\boldsymbol{e}}{\boldsymbol{i}}{\boldsymbol{g}}{\boldsymbol{h}}{\boldsymbol{t}}}\times 100$$

#### Proline

Proline content was determined according to the method described by Bates et al. (1973).

#### Antioxidant enzyme activities

Ascorbate peroxidase (APX) activity (mg^−1^ protein) was estimated according to the method of Nakano and Asada (1981)^21^ and catalase (CAT) activity (mg^−1^ protein) was determined according to Rao et al. (1997)^22^.

#### Soil enzyme activities

##### Dehydrogenase activity

Dehydrogenase (DHA) activity (mg TPF g^−1^ soil day^−1^) in the soil was estimated according to Casida et al. (1964)^23^. Urease activity (mg NH_4_^+^-N g^−1^ soil day^−1^) was measured according to the method described by Pancholy and Rice (1973)^24^.

#### Measurement of N, K, Na and the K/Na ratio in cowpea leaves

Plant leaves were digested according to the method described by Campbell and Plank (1998)^25^. Nitrogen content (mg plant^−1^) was determined using the micro-Kjeldahl method^26^. Na^+^, K^+^, and K^+^/Na^+^ ratio were determined using a flame photometer according to Page et al. (1982)^27^.

#### Cowpea productivity

The number of pods plant^−1^, 100-seed weight, and yield (t ha^−1^) were estimated four months after sowing.

#### Statistical analysis

CoStat software (Package 6.45, CoHort, USA) was used for statistical analysis of the data in accordance with the analysis of variance (ANOVA) approach. The full ANOVA (mixed-model) specification included irrigation intervals and foliar spray as fixed factors. The random factors were replicates/blocks and the replicate × irrigation interval interaction. Including replicate (or block) as a random effect is essential, as this is standard practice in split-plot field experiments. Omitting it would lead to incorrect F-tests and inflated significance levels. Duncan’s multiple range test (DMRT) was used to compare treatment means at p < 0.05^28^. Data are presented as means ± standard deviation (SD).

## Results

### Identification *AE_3 *by *rpoC1* gene analysis

**Fig. 1:**
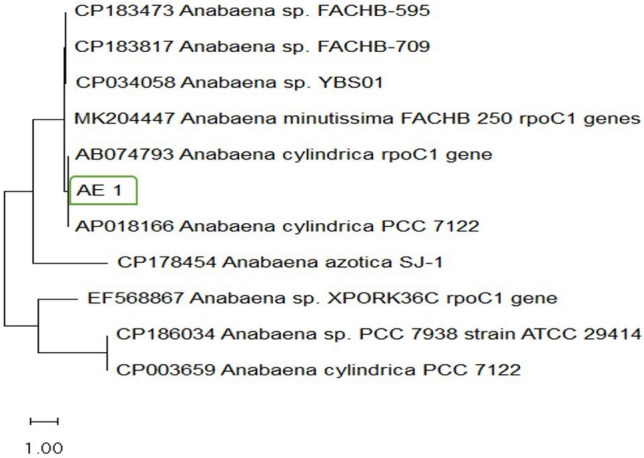
A phylogenetic tree based on the *rpoC1* gene sequence analysis showing the phylogenetic position of isolate, AE_3 (green box) among related *Anabaena* spp. retrieved from the GenBank database. Bootstrap values (%) based on 1000 replicates are shown at branch nodes.

The *rpoC1* gene sequence of isolate AE_3 was compared with related sequences available in GenBank database using BLAST analysis. The obtained sequence showed 96.04% similarity to *Anabaena cylindrica* (Table 5). Phylogenetic analysis revealed that isolate AE_3 clustered closely with *Anabaena cylindrica* sequences retrieved from GenBank, confirming its phylogenetic affiliation with the genus *Anabaena* (Fig. 1).

**Table 5:** rpoC1 gene sequences of related samples with similarity percentages of more than 96.04%, downloaded from the GenBank database.

Isolate	Accession number	E-value	Query coverage%	Similarity %
*Anabaena cylindrica*	AB074793.1	0.0	100	96.04
*Anabaena* sp*.*	CP186034.1	0.0	100	96.04
*Anabaena cylindrica*	CP003659.1	0.0	100	96.04
*Anabaena cylindrica*	AP018166.1	0.0	100	96.04
*Anabaena minutissima*	MK204447.1	0.0	99	90.01
*Anabaena azotica*	CP178454.1	0.0	99	90.01
*Anabaena* sp	EF568867.1	0.0	100	84.86
*Anabaena* sp	CP183473.1	0.0	100	84.80
*Anabaena* sp	CP183817.1	0.0	100	84.80
*Anabaena* sp	CP034058.1	0.0	99	84.27

Accordingly, the results of the phylogenetic tree sequence analysis of *AE-3* with the sequence of alignment on database (Fig 1), showing two main clusters;

### GC–MS analysis of metabolites produced by *Bradyrhizobium *and *Anabaena*

GC- MS analysis show the presence of several bioactive compounds in *Bradyrhizobium* including, formic acid (36.35%),- (+)- Ascorbic acid 2,6-dihexadecanoate (5.68%), Benzoic acid, 3.5 – dimethyl-, methyl ester (3.31%),3,5 – di- t- Butvicatechol (2.06%), Phenol, 4-tert- butyl (1.3%), Rapamycin (0.64%), and Vanillin (0.45%). Also, *A. cylinderica* enriched in n-Hexadecanoic acid (7.09%), phthalic acid (3.72%), Isopropyl linoleate (3.11%), Vanillin lactoside (0.58%), Phenol (0.7%), Quinoline, decahydro-, Oleic Acid (0.45%), cis-Vaccenic acid (0.59%), cis-13-Eicosenoic acid (0.3%), and Trilinolein (0.15%). Compound identification was based on relative peak area (%) of the total ion chromatogram (TIC), without internal standard normalization.

### Plant growth substances of *Anabaena cylindrica*

**Fig. 2:**
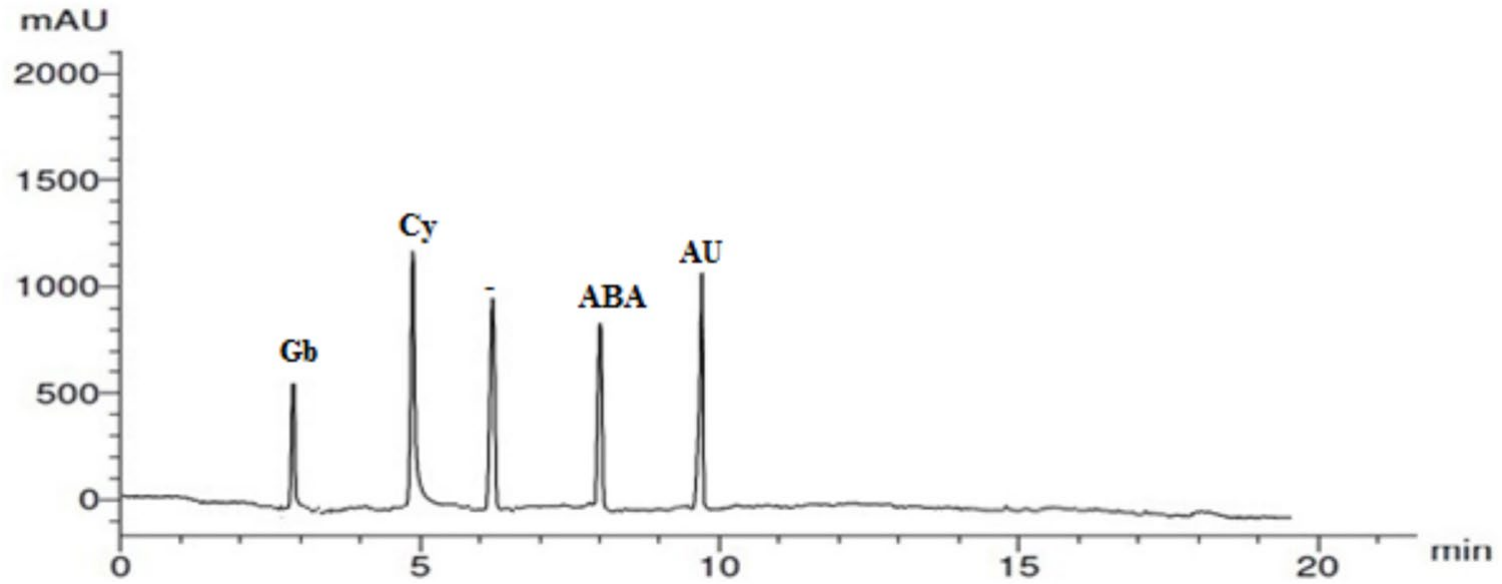
Plant growth substances of *Anabaena cylindrical.*

HPLC analysis show that the total hormones produce by *Anabaena cylindrica *are, gebrellic acid (Gb) (6.10 μg/gm), cytokinin (Cy) (13.23 μg/gm), auxin (Au) (9.06 μg/gm), and absisic acid 7.25 μg/gm) (Fig. 2).

### Effect of foliar spraying with *Bradyrhizobium *and* Anabaena* on physiological and biochemical properties of cowpea under irrigation intervals.

The effects of irrigation interval, foliar spraying, and their interaction on total chlorophyll, carotenoids, TSS, RWC, and proline accumulation in cowpea leaves were significant during both the 2022 and 2023 seasons (P < 0.05) are presented in Tables 6 and 7. Total chlorophyll and carotenoids showed a maximum value with a mixture of *Bradyrhizobium* plus *Anabaena* under treatment T_10_, recording 21.14 and 2.49 mg g^−1^ FW in 2022 and 22.36 and 2.56 mg g^−1^ FW in 2023, respectively. It was also noted that, the longest irrigation interval (T_3_) recorded the lowest value of chlorophyll and carotenoid contents in two seasons. The same trend was observed for TSS and RWC. Under T_10_ (consortium with irrigation every 10 days) yielded the highest value (7.72 and 7.81 µg g^−1^ FW) and (78.62% and 81.32%), respectively, during the two seasons. Under water deficit, all treatments exposed to water stress (T_3_, T_6_, T_9_, and T_12_) gave lower values ​​compared to the other treatments, but the treatments containing *Bradyrhizobium* and* Anabaena*, either individually or in combination, improved plant tolerance to water stress. Conversely, the proline content showed the opposite trend, with the highest concentration under water shortage (T_3:_ 9.16 and 9.25 µmol g^−1^ FW) in two seasons, respectively. When compared to the stressed control, foliar application of *Bradyrhizobium* or *Anabaena* decreased proline content, but T_10_ had the lowest proline content of any treatment. Longer irrigation intervals (15 and 18 days) significantly reduced RWC %, carotenoids, total soluble sugars (TSS), and total chlorophyll content. Irrigation after 18 days (T_3_), resulted in the lowest values of the measured physiological parameters, indicating the strongest adverse effects water stress. This stress adaptation response was accompanied by a significant increase in proline content. Although, the adverse effects of water stress were alleviated by the application *Anabaena* and *Bradyrhizobium* or consortium under 15 days irrigation interval. In comparison to the control (T_2_), treatments T5, T8, and particularly T_11_ improved (RWC) and total chlorophyll content while reducing proline accumulation. *Bradyrhizobium* and *Anabaena* (T_6_, T_9_, and T_12_) helped to improve these parameters to some degree under more water stress conditions (irrigation every 18 days). The consortium treatment (T_12_) shows the greatest ability to mitigate the negative effects of water stress compared with T_3_. These findings suggest that using *Bradyrhizobium* and *Anabaena*, or in a consortium, represents a promising strategy for reducing drought-induced damage and enhancing cowpea tolerance irrigation to prolonged irrigation intervals.

**Table 6:** Combined effects of different irrigation intervals and foliar spray with cultures of *Bradyrhizobium* and *Anabaena* on total chlorophyll, carotenoids, total soluble sugars (TSS) in cowpea leaves at 50 days after sowing during 2022 and 2023 seasons.

Treatment	Total chlorophyll (mg g^−1^ FW)		Carotenoids (µg g^−1^ FW)		Total soluble sugars (TSS) (µg g^−1^ FW)	
	2022	2023	2022	2023	2022	2023
T_1_	17.38 ± 1.09 ^d^	18.60 ± 1.11 ^d^	1.94 ± 0.22 ^d^	2.01 ± 0.31 ^d^	6.01 ± 0.66 ^d^	6.10 ± 0.67 ^d^
T_2_	14.62 ± 0.96 ^gh^	15.60 ± 1.18 ^hi^	1.60 ± 0.25 ^gh^	1.64 ± 0.27 ^hi^	4.97 ± 0.61 ^gh^	5.02 ± 0.63 ^gh^
T_3_	14.08 ± 1.01 ^h^	15.16 ± 1.21 ^i^	1.55 ± 0.29 ^h^	1.60 ± 0.21 ^i^	4.79 ± 0.71 ^gh^	4.86 ± 0.72 ^h^
T_4_	18.93 ± 1.09 ^c^	20.15 ± 1.27 ^c^	2.11 ± 0.31 ^c^	2.18 ± 0.19 ^c^	6.54 ± 0.77 ^c^	6.63 ± 0.70 ^c^
T_5_	15.39 ± 1.13 ^ef^	16.37 ± 1.09 ^efg^	1.69 ± 0.33 ^ef^	1.73 ± 0.15 ^efg^	5.24 ± 0.62 ^ef^	5.29 ± 0.68 ^ef^
T_6_	14.35 ± 0.86 ^gh^	15.43 ± 1.11 ^hi^	1.58 ± 0.22 ^gh^	1.63 ± 0.22 ^hi^	4.88 ± 0.55 ^gh^	4.95 ± 0.79 ^gh^
T_7_	20.25 ± 1.21 ^b^	21.47 ± 1.27 ^b^	2.38 ± 0.28 ^b^	2.45 ± 0.15 ^b^	7.38 ± 0.58 ^b^	7.47 ± 0.66 ^b^
T_8_	15.57 ± 1.11 ^ef^	16.55 ± 0.99 ^ef^	1.71 ± 0.19 ^ef^	1.75 ± 0.22 ^ef^	5.30 ± 0.71 ^e^	5.35 ± 0.55 ^ef^
T_9_	14.60 ± 0.92 ^gh^	15.68 ± 1.04 ^ghi^	1.60 ± 0.18 ^gh^	1.65 ± 0.26 ^ghi^	4.97 ± 0.61 ^gh^	5.04 ± 0.59 ^gh^
T_10_	21.14 ± 1.25 ^a^	22.36 ± 1.09 ^a^	2.49 ± 0.21 ^a^	2.56 ± 0.28 ^a^	7.72 ± 0.68 ^a^	7.81 ± 0.67 ^a^
T_11_	15.89 ± 1.03 ^e^	16.87 ± 1.01 ^e^	1.74 ± 0.29 ^e^	1.78 ± 0.31 ^e^	5.41 ± 0.77 ^e^	5.46 ± 0.62 ^e^
T_12_	14.86 ± 0.91 ^fg^	15.94 ± 1.07 ^fgh^	1.63 ± 0.22 ^fg^	1.68 ± 0.27 ^fgh^	5.06 ± 0.58 ^fg^	5.13 ± 0.57 ^fg^
**F-test**						
Main	**	**	**	**	**	**
Sub main	**	**	**	**	**	**
Interaction	**	**	**	**	**	**

Means followed by different letters indicate significant differences among treatments according to the Duncan’s test (P < 0.05). Values are means ± standard deviation (SD) from 3 replicates (Means ± SD).

**Table 7:** Combined effects of different irrigation intervals and foliar application with cultures of *Bradyrhizobium* and *Anabaena* on Relative Water Content (RWC) (%) and proline contents in cowpea leaves at 50 days after sowing during 2022 and 2023 seasons.

Treatment	Relative water content (RWC) (%)		Proline (µmole g^−1^ FW)	
	2022	2023	2022	2023
T_1_	71.35 ± 3.89 ^d^	74.05 ± 3.21 ^d^	6.50 ± 0.58 ^fg^	6.58 ± 0.78 ^fgh^
T_2_	65.77 ± 3.21 ^fg^	68.07 ± 3.09 ^h^	6.85 ± 0.61 ^e^	6.90 ± 0.82 ^e^
T_3_	63.38 ± 2.99 ^h^	66.28 ± 2.89 ^i^	9.16 ± 0.66 ^a^	9.25 ± 0.88 ^a^
T_4_	73.17 ± 3.45 ^c^	75.87 ± 2.91 ^c^	6.41 ± 0.59 ^gh^	6.49 ± 0.83 ^ghi^
T_5_	69.27 ± 2.89 ^e^	71.57 ± 2.96 ^f^	6.74 ± 0.71 ^e^	6.79 ± 0.79 ^ef^
T_6_	64.58 ± 3.27 ^gh^	67.48 ± 3.11 ^hi^	8.82 ± 0.69 ^b^	8.91 ± 0.65 ^b^
T_7_	76.10 ± 3.33 ^b^	78.80 ± 3.34 ^b^	6.32 ± 0.63 ^gh^	6.40 ± 0.58 ^hi^
T_8_	70.06 ± 3.09 ^de^	72.36 ± 3.14 ^ef^	6.68 ± 0.67 ^ef^	6.73 ± 0.59 ^efg^
T_9_	65.71 ± 2.96 ^fg^	68.61 ± 2.91 ^gh^	7.98 ± 0.55 ^c^	8.07 ± 0.76 ^c^
T_10_	78.62 ± 2.88 ^a^	81.32 ± 3.31 ^a^	6.23 ± 0.78 ^h^	6.31 ± 0.84 ^i^
T_11_	71.51 ± 3.01 ^d^	73.81 ± 2.94 ^de^	6.41 ± 0.81 ^gh^	6.46 ± 0.72 ^hi^
T_12_	66.87 ± 3.08 ^f^	69.77 ± 3.15 ^g^	7.45 ± 0.73 ^d^	7.54 ± 0.79 ^d^
**F-tes**t				
Main	**	**	**	**
Sub main	**	**	**	**
Interaction	**	**	**	**

Means followed by different letters indicate significant differences among treatments according to the Duncan’s test (P < 0.05). Values are means ± standard deviation (SD) from 3 replicates (Means ± SD).

See Table 2 for treatment details

See Table 2 for treatment details

### Effect of foliar application with *Bradyrhizobium *and* Anabaena* on the number and dry weight of cowpea nodules under irrigation intervals.

**Fig. 3:**
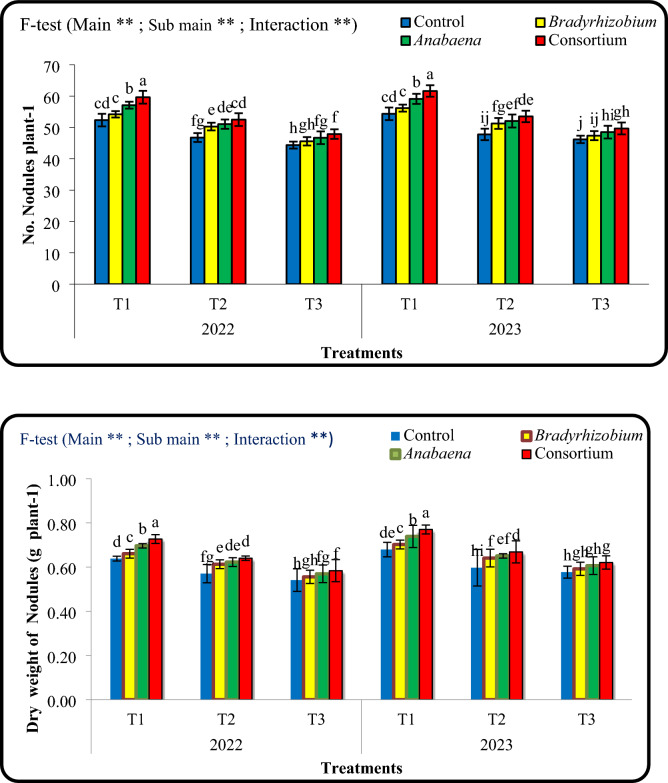
Combined effects of different irrigation intervals and foliar spray with cultural of Bradyrhizobium , Anabaena, and consortium on number of nodules and dry weight if nodules (g plant-1) in cowpea leaves at 50 days from sowing during 2022 and 2023 seasons. Means followed by different letters indicate significant differences among treatments according to the Duncan’s test (P < 0.05). Values are means ± standard deviation (SD) from 3 replicates (Means ± SD).

The results in Fig. 3 show that the consortium or *Anabaena* significantly increased nodule formation compared with the control and *Bradyrhizobium* alone in two seasons. The consortium treatment recorded the highest number of nodules, highlighting the combination effect of *Anabaena* and *Bradyrhizobium* in enhancing nodulation and potentially improving nitrogen fixation. Nodule number decreased under the longer irrigation intervals (T_2_ and T_3_) compared with T_1_. Treatments involving the consortium or *Anabaena* maintained higher nodule numbers during two seasons*.* Similar trends were observed for nodule dry weight, where the consortium treatment produced the highest values under all irrigation conditions. The positive impact of microalgae-based biofertilization on nodulation is stable under different conditions.

### Effect of foliar application with *Bradyrhizobium* and* Anabaena* on N, K, Na and K/Na % in cowpea leaves at 50 days after sowing during 2022 and 2023 seasons.

The data in Table 8 showed that foliar spraying with *Bradyrhizobium* and *Anabaena*, particularly treatment T_10_, improved the leaf nitrogen (N) and potassium (K) contents and increased the K^+^/Na^+^ ratio compared to other treatments. Under water deficit conditions (T_2_ and T_3_), nitrogen and potassium contents decreased, while sodium accumulation increased considerably, especially in treatment T_3_. Treatments containing *Bradyrhizobium* and *Anabaena*, especially the treatment combined application, improved leaf content of nitrogen and potassium and reduced sodium accumulation under water stress conditions during the 2022 and 2023 seasons.

**Table 8:** Combined effects of different irrigation intervals and foliar application with cultures of *Bradyrhizobium* and *Anabaena* on N, K, Na and K/Na ratio in cowpea leaves at 50 days after sowing during 2022 and 2023 seasons.

Treatment	N (mg DW^−1^)		K%		Na%		K/Na ratio	
	2022	2023	2022	2023	2022	2023	2022	2023
T_1_	2.32 ± 0.19 ^d^	2.44 ± 0.17 ^d^	1.21 ± 0.11 ^d^	1.29 ± 0.05 ^d^	1.40 ± 0.15 ^fg^	1.51 ± 0.27 ^e-h^	0.86 ± 0.08 ^d^	0.85 ± 0.05 ^d^
T_2_	1.98 ± 0.31 ^gh^	2.06 ± 0.22 ^hi^	0.87 ± 0.09 ^gh^	0.92 ± 0.17 ^f^	1.51 ± 0.22 ^e^	1.58 ± 0.22 ^e^	0.58 ± 0.05 ^g^	0.58 ± 0.03 ^g^
T_3_	1.93 ± 0.22 ^h^	2.06 ± 0.28 ^hi^	0.82 ± 0.14 ^h^	0.85 ± 0.11 ^g^	2.26 ± 0.20 ^a^	2.38 ± 0.18^a^	0.36 ± 0.04 ^i^	0.36 ± 0.02 ^j^
T_4_	2.49 ± 0.28 ^c^	2.61 ± 0.25 ^c^	1.38 ± 0.13 ^c^	1.46 ± 0.19 ^c^	1.37 ± 0.19 ^gh^	1.48 ± 0.19 ^f-i^	1.00 ± 0.11 ^c^	0.98 ± 0.09 ^c^
T_5_	2.07 ± 0.18 ^ef^	2.15 ± 0.31 ^efg^	0.96 ± 0.17 ^ef^	1.01 ± 0.07 ^e^	1.48 ± 0.16 ^ef^	1.55 ± 0.22 ^ef^	0.65 ± 0.09 ^f^	0.65 ± 0.06 ^f^
T_6_	1.96 ± 0.26 ^gh^	2.09 ± 0.27 ^ghi^	0.85 ± 0.11 ^gh^	0.88 ± 0.09 ^fg^	2.15 ± 0.22 ^b^	2.27 ± 0.27 ^b^	0.39 ± 0.04 ^i^	0.39 ± 0.03 ^j^
T_7_	2.76 ± 0.20 ^b^	2.88 ± 0.18 ^b^	1.65 ± 0.06 ^b^	1.73 ± 0.21 ^b^	1.35 ± 0.16 ^gh^	1.46 ± 0.15 ^ghi^	1.23 ± 0.13 ^b^	1.19 ± 0.11 ^b^
T_8_	2.09 ± 0.21 ^ef^	2.17 ± 0.13 ^ef^	0.98 ± 0.14 ^ef^	1.03 ± 0.11 ^e^	1.46 ± 0.23 ^ef^	1.53 ± 0.17 ^efg^	0.67 ± 0.08 ^f^	0.67 ± 0.07 ^f^
T_9_	1.98 ± 0.25 ^gh^	2.11 ± 0.26 ^f-i^	0.87 ± 0.16 ^gh^	0.90 ± 0.06 ^fg^	1.88 ± 0.19 ^c^	2.00 ± 0.27 ^c^	0.47 ± 0.02 ^h^	0.45 ± 0.05 ^i^
T_10_	2.87 ± 0.11 ^a^	2.99 ± 0.28 ^a^	1.76 ± 0.17 ^a^	1.84 ± 0.17 ^a^	1.32 ± 0.26 ^h^	1.43 ± 0.18 ^i^	1.34 ± 0.12 ^a^	1.29 ± 0.12 ^a^
T_11_	2.12 ± 0.17 ^e^	2.20 ± 0.14 ^e^	1.01 ± 0.12 ^e^	1.06 ± 0.10 ^e^	1.37 ± 0.32 ^gh^	1.44 ± 0.29 ^hi^	0.74 ± 0.08 ^e^	0.74 ± 0.08 ^e^
T_12_	2.01 ± 0.19 ^fg^	2.14 ± 0.17 ^e-h^	0.90 ± 0.09 ^fg^	0.93 ± 0.13 ^f^	1.71 ± 0.30 ^d^	1.83 ± 0.15 ^d^	0.53 ± 0.09 ^gh^	0.51 ± 0.09 ^h^
**F-test**								
Main	**	**	**	**	**	**	**	**
Sub main	**	**	**	**	**	**	**	**
Interaction	**	**	**	**	**	**	**	**

Means followed by different letters indicate significant differences among treatments according to the Duncan’s test (P < 0.05). Values are means ± standard deviation (SD) from 3 replicates (Means ± SD).

See Table 2 for treatment details

### Effect of foliar application with *Bradyrhizobium *and* Anabaena* on the activities of catalase (CAT) and ascorbate peroxidase (APX) enzymes during 2022 and 2023 seasons.

**Fig. 4:**
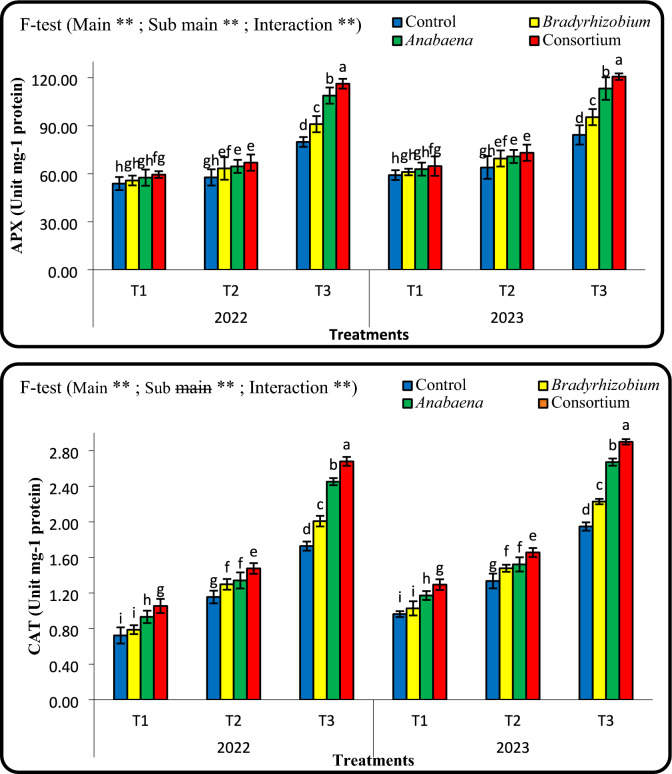
Combined effects of different irrigation intervals and foliar spray with cultural of Bradyrhizobium , Anabaena, and consortium on ascorbate peroxidase (APX) and catalase (CAT) activities in cowpea leaves at 50 days from sowing during 2022 and 2023 seasons. Means followed by different letters indicate significant differences among treatments according to the Duncan’s test (P < 0.05). Values are means ± standard deviation (SD) from 3 replicates (Means ± SD).

In cowpea leaves, foliar spraying of *Bradyrhizobium* and *Anabaena*, as well as irrigation intervals significantly affected catalase (CAT) and ascorbate peroxidase (APX) activities (Fig.4). Both CAT and APX enzymes exhibited relatively low activity under well irrigation conditions (T1), which indicated a low level of oxidative stress. However, CAT and APX activities were significantly increased under water-deficit conditions (T_3_), indicating that these two enzymes play an important role in the scavenging of ROS generated during drought stress. The highest CAT and APX activities were recorded in the consortium, followed by *Anabaena* alone, while untreated control consistently showed the lowest values. The trend indicates that *Bradyrhizobium* and *Anabaena* enhance the antioxidant defense system of cowpea under drought stress conditions.

### Effect of foliar spraying with *Bradyrhizobium *and* Anabaena* on dehydrogenase (DHA) and urease enzyme activities during 2022 and 2023 seasons.

**Fig. 5:**
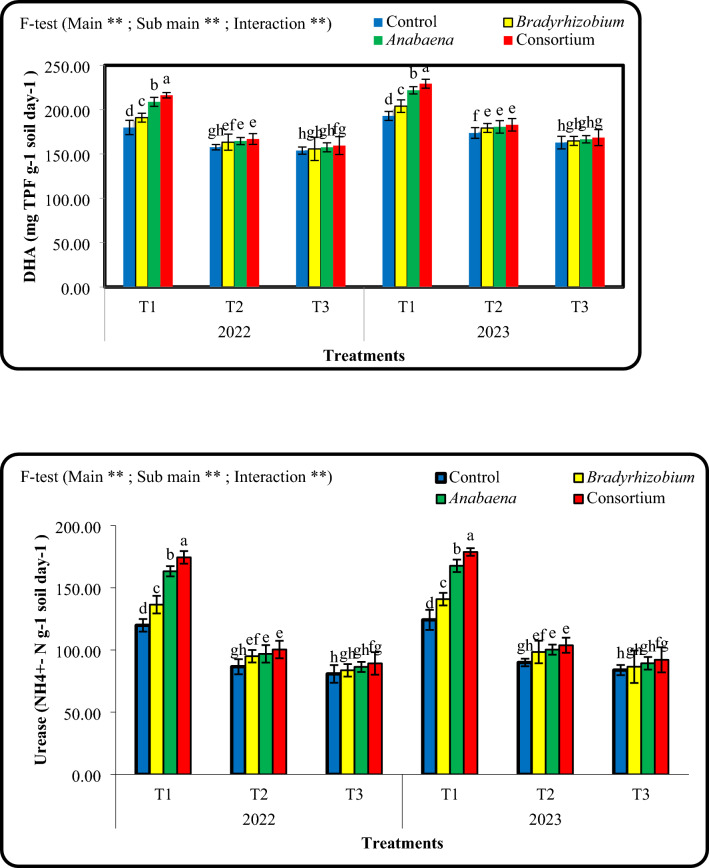
Combined effects of different irrigation intervals and foliar spray with cultural of Bradyrhizobium, Anabaena and consortium on soil enzymes activity (dehydrogenase (DHA), and urease) in the rhizosphere of cowpea plants at 50 days from sowing during 2022 and 2023 seasons. Means followed by different letters indicate significant differences among treatments according to the Duncan’s test (P < 0.05). Values are means ± standard deviation (SD) from 3 replicates (Means ± SD).

Foliar spraying of *Bradyrhizobium* and *Anabaena* and irrigation intervals, significantly affected dehydrogenase (DHA) and urease activities in the rhizosphere of cowpea plants (Fig. 5). The irrigation intervals of 10 days resulted in significantly higher enzyme activities, while there was a gradual decrease in the enzyme activity with prolonged irrigation intervals for 15 and 18 days, particularly with irrigation after 18 days interval. On the other hand, *Bradyrhizobium*, *Anabaena*, and a combination, partially alleviated the negative effects of water scarcity on soil enzymatic activity. The highest urease and DHA activities were observed under irrigation every 10 days, particularly in the combined *Bradyrhizobium* + *Anabaena* treatment, whereas extending the irrigation interval to 15 or 18 days significantly reduced enzyme activity during both growing seasons.

### Effect of foliar spraying with *Bradyrhizobium *and* Anabaena* on yield, 100-seed weight, and number of pods during 2022 and 2023 seasons.

The results in Table 9 demonstrate that the cowpea yield and its components were significantly affected by irrigation intervals and the microbial foliar treatment during the 2022 or 2023 seasons. Under 10-day irrigation interval, cowpea plants showed the highest values for yield and yield components. Increasing the irrigation interval to 15 or 18 days (T_2_ and T_3_) caused a negative effect of water stress on flowering and pod setting and filling of seeds, inducing remarkable reductions in all yield parameters. Foliar application of Bradyrhizobium (T_4_–T_6_) and *Anabaena* (T_7_–T_9_) partially alleviated the adverse effects of water stress with best performance under the 10 days irrigation interval (T_4_ and T_7_). The consortium treatments (T_11_ and T_12_) significantly outperformed the corresponding untreated controls (T_2_ and T_3_) and the individual microbial treatments under 15 and 18 days intervals. Higher yield qualities were reported by *Bradyrhizobium* (T_5_ and T_6_) and *Anabaena* (T_8_ and T_9_) treatments as compared to their respective controls, suggesting a partial alleviation of water stress. The consortium treatments (T_11_ and T_12)_ showed the greatest improvement under both 15 and 18-day intervals, surpassing the single treatments. The presence of both *Bradyrhizobium* and *Anabaena* resulted in an estimated 33% increase in yield when irrigated every 15 days and more than 23% when irrigated every 18 days compared to the control.

**Table 9:** Combined effects of different irrigation intervals and foliar spray with cultures of *Bradyrhizobium* and *Anabaena* on number of pods (plant^−1^), 100-seed weight (g) and yield (ton ha^−1^) in cowpea leaves at 50 days after sowing during 2022 and 2023 seasons.

Treatment	Number of pods plant^−1^		100 – seed weight (g)		Yield (ton ha^−1^)	
	2022	2023	2022	2023	2022	2023
T_1_	22.35 ± 1.30 ^cd^	25.35 ± 1.28 ^de^	19.35 ± 1.23 ^d^	19.43 ± 1.02 ^cd^	3.72 ± 0.88 ^d^	3.92 ± 0.12 ^d^
T_2_	16.77 ± 1.20 ^fg^	20.77 ± 1.87 ^f^	13.77 ± 1.19 ^fg^	13.83 ± 1.02 ^fg^	2.80 ± 0.34 ^fg^	2.85 ± 0.22 ^gh^
T_3_	14.38 ± 1.50 ^h^	17.38 ± 1.28 ^h^	11.38 ± 1.09 ^h^	11.42 ± 1.11 ^h^	2.40 ± 0.28 ^h^	2.47 ± 0.24 ^i^
T_4_	24.17 ± 1.90 ^c^	27.17 ± 2.01 ^c^	21.17 ± 1.11 ^c^	21.25 ± 1.19 ^c^	4.03 ± 0.32 ^c^	4.23 ± 0.55 ^c^
T_5_	20.27 ± 2.00 ^e^	24.27 ± 1.98 ^e^	17.27 ± 1.09 ^e^	17.33 ± 1.23 ^e^	3.38 ± 0.39 ^e^	3.43 ± 0.29 ^f^
T_6_	15.58 ± 1.00 ^gh^	18.58 ± 1.46 ^gh^	12.58 ± 1.02 ^gh^	12.62 ± 1.31 ^gh^	2.60 ± 0.27 ^gh^	2.67 ± 0.33 ^hi^
T_7_	27.10 ± 1.70 ^b^	30.10 ± 1.28 ^b^	24.10 ± 1.02 ^b^	24.18 ± 1.21 ^b^	4.52 ± 0.34 ^b^	4.72 ± 0.40 ^b^
T_8_	21.06 ± 2.01 ^de^	25.06 ± 2.09 ^de^	18.06 ± 1.12 ^de^	18.12 ± 1.11 ^de^	3.51 ± 0.22 ^de^	3.56 ± 0.28 ^ef^
T_9_	16.71 ± 1.98 ^fg^	19.71 ± 1.86 ^fg^	13.71 ± 1.08 ^fg^	13.75 ± 1.31 ^fg^	2.78 ± 0.29 ^fg^	2.85 ± 0.18 ^gh^
T_10_	29.62 ± 2.10 ^a^	32.62 ± 2.11 ^a^	26.62 ± 1.87 ^a^	26.70 ± 1.27 ^a^	4.94 ± 0.34 ^a^	5.14 ± 0.65 ^a^
T_11_	22.51 ± 1.92 ^cd^	26.51 ± 1.92 ^cd^	19.51 ± 1.12 ^d^	19.57 ± 1.22 ^cd^	3.75 ± 0.17 ^d^	3.80 ± 0.23 ^de^
T_12_	17.87 ± 1.55 ^f^	20.87 ± 1.76 ^f^	14.87 ± 1.04 ^f^	14.91 ± 1.15 ^f^	2.98 ± 0.19 ^f^	3.05 ± 0.48 ^g^
**F-test**						
Main	**	**	**	**	**	**
Sub main	**	**	**	**	**	**
Interaction	**	**	**	**	**	**

Means followed by different letters indicate significant differences among treatments according to the Duncan’s test (P < 0.05). Values are means ± standard deviation (SD) from 3 replicates (Means ± SD).

See Table 2 for treatment details

## Discussion

The study revealed a significant improvement in cowpea growth, physiological performance, and productivity under deficit irrigation conditions following foliar application of *Bradyrhizobium* sp. and *Anabaena cylindrica*, individually or in combination. Prolonged irrigation intervals of 15 and 18 days resulted in water stress, which adversely affected chlorophyll, carotenoids, total soluble sugars, and relative water content levels. Proline accumulation increased as an osmotic adjustment response to water stress, as previously reports related to legumes under water stress conditions^29^. The elevated proline content in T_3_ indicates enhanced osmotic adjustment under stress conditions, whereas microbial treatments significantly reduced proline accumulation, suggesting alleviation of stress-induced metabolic responses. Most importantly, foliar spray of the investigated microorganisms, particularly the consortium treatment, helped alleviate the negative effects of water stress by improving chlorophyll, carotenoids, RWC, TSS, while reducing proline accumulation compared to stress controls. This enhanced cowpea tolerance to water stress maintain productivity under limited irrigation conditions. GC-MS and HPLC analysis revealed the presence of several bioactive compounds produced by the microbes, including auxins, cytokinins, gibberellins, and abscisic acid. These compounds may have contributed to improved photosynthetic efficiency, water relations, and osmotic adjustment and hence allowed plants to better tolerate drought stress^30^**,** Arenas et al.^31^. The combined action of rhizobia and cyanobacteria could potentially enhance nitrogen fixation, develop roots, and thus improve nutrient uptake. All this would give rise to improved physiological properties, such as chlorophyll and enzymatic activities under a limited water^5,6,32,33^**.** The interaction between irrigation intervals and foliar application significantly regulated plant physiological responses under water-stress conditions. The supremacy of the consortium treatment (T_10_) reflects the effect of integrating water management strategy with *Bradyrhizobium* and *Anabaena* treatments. Under prolonged irrigation intervals, the reduced proline content in treatments containing *Bradyrhizobium* and *Anabaena* showed a mitigation of drought stress because proline accumulation is considered one of the well-known physiological indices of plant stress. This response was associated with improved intracellular water balance and reduced oxidative damage, which positively influenced plant physiological status and irrigation water use efficiency. Consequently, these treatments enhanced plant tolerance to prolonged irrigation intervals application of *Bradyrhizobium* and *Anabaena* significantly increased root nodule formation in the formation compared with the control and *Bradyrhizobium*- alone treatments during both growing seasons. This effect may be attributed to the presence of organic compounds, plant growth regulators, amino acids, vitamins, and antioxidants, which improve plant health, increase root cell division, stimulate the formation of effective bacterial nodules, and increase the efficiency of atmospheric nitrogen fixation. The dry weight of root nodules followed a similar trend, confirming that the increase was not limited to nodule number, but also in the functional efficiency and nodule biomass. The positive effects of the microalgae and consortium treatments were consistently observed under different irrigation conditions. Nutrient analysis revealed that treatments with *Bradyrhizobium* and *Anabaena* increased leaf nitrogen and potassium contents while reducing sodium accumulation, resulting in improved K/Na ratios. Enhanced potassium uptake likely contributed to improve stomatal regulation and water use efficiency, while reduced sodium accumulation mitigated ion toxicity under deficit irrigation conditions, , Arenas et al.^31,34^. Hence, foliar application of *Bradyrhizobium* and *Anabaena* consortium may represent an effective strategy for alleviating water and salinity stresses while improving plant nutrition and ionic balance during the 2022 and 2023 seasons. Moreover, cowpea plants treated with the consortium with *Anabaena* alone showed the highest CATand APX activities. This response may be attributed to the ability of these microbes to produce bioactive compound, thereby increasing plant physiological efficiency under stress conditions and improving nitrogen and mineral uptake. A gradual reduction in dehydrogenase and urease enzyme activities was observed with decreasing soil moisture content under prolonged irrigation intervals, particularly under the 18-day irrigation interval (T_3_), although the microbial application partially alleviated the adverse effects of water scarcity^35,36^.These finding further revealed the ability of the microbial application to stimulate soil microbial activity through the release of growth-promoting substances, including hormones, organic acids, and enzymes thereby enhancing plant and soil tolerance to water stress. Yield components, including number of pods, 100-seed weight, and total seed yield, were significantly higher in plants treated with the microbial consortium, even under extended irrigation intervals. This enhancement may be attributed to improved root growth, nutrient and water uptake, biological nitrogen fixation, and improved hormonal regulation involved in flowering and seed production^7,37^. Reproducibility of these beneficial effects under variable field conditions and different soil types requires further validation through multi-location trial. This study has some limitations, including evaluation at a single location and on only one cowpea cultivar. Furthermore, microbial persistence and colonization on leaf surfaces were not assessed. Therefore, further studies under diverse environmental conditions are needed to validate the broader applicability of the obtained results. The results of the study reveal that foliar application of a rhizobial-cyanobacterial consortium represents a promising and environmentally friendly approach to improve cowpea tolerance to water-deficit stress. The treatment enhanced physiological and biochemical attributes, stimulated rhizosphere enzymatic activity, and stabilize productivity under water- deficit conditions. The results provide strong support for the practical application of microbial consortia in sustainable agriculture under water-limited conditions.

## Conclusion

This study showed that foliar application of a consortium of *Bradyrhizobium* sp. and *Anabaena cylindrica* improved cowpea growth, performance, and productivity under water deficit conditions. The combined treatment increased chlorophyll and carotenoid content, total soluble sugars, and relative water content, along with a significant reduction in proline accumulation under prolonged irrigation intervals, suggesting alleviation of drought-induced stress. Higher activities of soil urease, dehydrogenase, CAT, and APX enzymes were observed under the consortium treatment, indicating enhanced soil biological activity and antioxidant defense responses. Additionally, the treatment enhanced leaf nitrogen and potassium contents, reduced sodium accumulation, and promoted root nodulation and dry matter accumulation. Even under prolonged irrigation intervals (15 and 18 days), the consortium treatment maintained a higher K/Na ratio and cowpea yield components,, including pod number, 100-seed weight, and total yield (ha^-1^). These results demonstrate that the integration of *Bradyrhizobium* and *A. cylindrica* may contribute to improve water and nutrient use efficiency and enhance cowpea performance and moderate water stress conditions. Further studies are recommended to evaluate the economic feasibility and large-scale performance of this microbial consortium under diverse field conditions.

## Data Availability

The datasets generated and/or analyzed during the current study are available from the corresponding author on reasonable request.

## Declarations

We agree to the participation and ethics
